# Germ-line transmission of trisomy 21: Data from 80 families suggest an implication of grandmaternal age and a high frequency of female-specific trisomy rescue

**DOI:** 10.1186/1755-8166-3-7

**Published:** 2010-03-18

**Authors:** Natalia V Kovaleva

**Affiliations:** 1 Scientific Research Centre at Saint-Petersburg State Pediatric Medical Academy under the Federal Agency of Health Care and Social Development, St Petersburg, Russian Federation

## Abstract

**Background:**

Trisomy of chromosome 21 (T21; Down syndrome, DS) is the most common aneuploidy in live births. Though its etiology has been intensively studied for a half of century, there are surprisingly many problems awaiting their elucidation. Some of the open questions are related directly to germ line mosaicism for T21, other problems include the prevalence of males with non-mosaic trisomy over females (skewed sex ratio, SR), the genetic predisposition to non-disjunction, etc. Studies in families of gonadal mosaicism (GM) carriers might help resolving some of these problems.

**Results:**

80 families of carriers of GM, in which the sex of the offspring had been specified, were identified in the literature and in logbooks of two local genetic units. Mothers in these families were relatively young: only 8% of mothers were 35 years old and older at the time of delivery of their first affected offspring while the proportion of grandmothers on the GM carrier's side aged 35 years old and older was significantly higher (39%). Postzygotic rescue of T21 due to error in the meiosis I had been proposed as a mechanism of parental GM formation in 78% of the families with known origin of the T21. For the other 22%, rescue of errors in the meiosis II or postzygotic mitotic non-disjunction was assumed. Mosaicism for T21 in successive generations was reported in at least 12 families. The proportion of mosaics among affected female offspring (14%) is significantly higher compared to that among affected male offspring (0%). Male preponderance (SR = 1.5) is found in non mosaic liveborn offspring with either maternally- or paternally transmitted T21. Among unaffected offspring of male carriers of GM there is a notable excess of females (SR = 0.27).

**Conclusion:**

Both direct (results of cytogenetic and molecular study of the origin of trisomic line) and indirect (advanced grandmaternal age on the side of GM carrier) evidences allow to assume that significant proportion of the mosaic parents had been conceived as trisomics. Female-specific trisomy rescue and genetic predisposition to postzygotic non-disjunction has been suggested as mechanisms of formation of both GM and somatic mosaicism. Typical male preponderance in affected non mosaic offspring with either maternally- or paternally transmitted trisomy 21, indicates than meiotic events are not responsible for the skewed sex ratio in DS. However a female excess among unaffected offspring of male carriers of GM might be the result of meiotic non homologous co-orientation of chromosomes 21 and X in spermatogenesis.

## Introduction

Trisomy of chromosome 21 (T21; Down syndrome, DS) is the most common aneuploidy in livebirths [1]. Though its etiology has been intensively studied for almost half a century, there are surprisingly many problems awaiting their elucidation [2]. Some of the open questions are related directly to germ line mosaicism (including precise evaluation the contribution of parental mosaicism to the occurrence of DS cases, the timing of mosaicism formation and the underlying mechanisms) [3,4]. Other problems are the genetic predisposition to non-disjunction (NDJ) [5], the effect of endogenous factors and environmental exposures, the prevalence of males with non-mosaic trisomy over females [6]. Studies in families of gonadal mosaicism (GM) carriers might help to resolve some of these problems.

Non-mosaic free T21 is mostly a result of NDJ in oogenesis. The majority of maternally derived trisomies occur in meiosis I, meiosis II errors only constitute about 20% of maternal errors. Paternally derived trisomies are several times less frequent and display about an equal number of errors in meiosis I and meiosis II. In about 4% of trisomic individuals, the additional chromosome appears to result from postzygotic error [7]. In a collective sample of 53 affected individuals with mosaic T21, somewhat different proportions were found, with a higher contribution of paternally derived extra chromosome (24%) and a higher rate (42%) of NDJ in meiosis II and mitotic NDJ in a normal euploid zygote [8]. Whether the contribution of different mechanisms to the formation of a trisomic line in asymptomatic GM carriers is different from that in affected individuals is still to be investigated.

There are few widely cited reports of mosaicism for T21 in two successive generations in one family [9-11] suggesting genetic predisposition to NDJ. Large samples are needed to confirm a genetic predisposition and to shed light on the underlying mechanism.

A study on the male to female ratio (sex ratio, SR) in individuals with maternally transmitted T21 might help to elucidate the phenomenon of the male prevalence among patients with DS. Male excess in DS patients with non-mosaic T21 is a well known but poorly understood phenomenon. The sex ratio varies in different populations, being close to average 1.3. Several hypotheses have been put forward to explain the skewed SR in DS. Meiotic disturbance (non-homologous co-orientation in male meiosis) [12,13], fertilization event (greater accessibility of Y-bearing sperm to ova disomic for chromosome 21 or promotion of non-disjunction in the ova by Y-bearing sperm) [14-16], and post-fertilization events (intrauterine selection against females) [17,18] have been discussed. Since females produce only X-bearing gametes, an approach to the problem may be the comparison of SR in cases of maternal and paternal transmission of T21.

Some earliest publications suggested that the risk for Down syndrome may be related to an aging grandmother [19,20]. Since then, not many studies on parental and gradparental ages in families of carriers of GM were reported. As expected, mean maternal age at birth of their DS children was found to be similar to that at the birth of a normal baby. However, the mean grandmaternal age at the birth of the mosaic parents was significantly higher than that at the birth of trisomic DS [21-23]. These studies supported a suggestion that most or all mosaic parents arose from trisomic zygotes [20]. To confirm this suggestion, more data on association of grandparental ages and grandparental origin of the mosaicism are needed.

The objectives of this study were: (1) to estimate the contribution of different mechanisms to the origin of germ line mosaicism; (2) to determine the prevalence of "inherited" mosaicism; (3) to study the sex ratio in affected offspring with respect to the mode of ascertainment and the parent of origin; (4) to study the sex ratio in unaffected offspring; and (5) to study maternal and grandmaternal ages in families of carriers of gonadal nosaicism.

## Results

### Families with maternal trisomy 21 gonadal mosaicism

Table S1 (see Additional file 1: Details of families with maternal trisomy 21 gonadal mosaicism) presents the data collected from reports on parental GM for T21. In total, 61 families were identified where the mother was a carrier of germ mosaicism, with 114 affected offspring for whom the sex was specified. Among the affected offspring, there were 50 males and 34 females (SR = 1.5) postnatally diagnosed as DS (including six patients with clinical diagnosis), 13 male and 12 female fetuses with prenatally diagnosed trisomy 21 (SR = 1.08), and 2 male and 3 female miscarried fetuses with T21. Six in 108 affected individuals/fetuses with confirmed T21 (5.6%) were found to be mosaic for a normal cell line. All of them were females. Among unaffected siblings, there were 19 males and 16 females.

In about half of the cases, maternal GM was inferred from the presence of a trisomic line in a single somatic tissue. In 15 cases, mosaicism found in blood cells was confirmed in cultured skin fibroblasts. In eight instances, a trisomic line was detected in an ovarian tissue, and in one case it was found in oocytes. In one remarkable case, maternal mosaicism was identified because of maternal cell contamination of an amniotic fluid specimen from a normal male pregnancy (Additional File 1: Table S1, case 53). In six cases, GM was uncovered by DNA analysis. Maternal origin of T21 in the affected offspring was proven in 15 families. In the majority of the informative cases (9/11), the study results were consistent with the original trisomy in the mother due to NDJ in first meiotic division followed by loss of one chromosome 21 (rescue of trisomy MI, for simplicity) as a mechanism of formation of germ-line mosaicism. In two families, the study results suggested that the origin of maternal mosaicism was either postzygotic mitotic or second meiotic division NDJ (rescue of trisomy MII). And in two families, the origin of maternal mosaicism was not clarified. In two nuclear families from the same pedigree (Additional File 1: Table S1, cases 2, 3) and in two other cases (Additional File 1: Table S1, cases 7, 61) the trisomy in the carriers was most probably inherited (rescue of transmitted trisomy).

Information on the maternal age at the time of delivery of their first affected offspring was available for 54 families. Two cases from surveys on prevalence of mosaicism in young parents (Additional File 1: Table S1, cases 14, 34) were excluded from the analysis of maternal age. The great majority of the mothers were young at the time of conception/delivery of their first affected offspring, the proportion of mothers aged 35 year and older was 3 out of 52 (5.8%).

### Families with paternal trisomy 21 gonadal mosaicism

Among 28 offspring in 19 families where the father was reported to be a carrier of T21 mosaicism (Additional File 2: Table S2, Details of families with paternal trisomy 21 gonadal mosaicism), there were 16 males and 10 females with postnatally diagnosed T21 (including one patient with clinical diagnosis), one prenatally detected female fetus, and one miscarried female fetus. Three out of 27 cytogenetically confirmed T21 cases displayed mosaicism for a normal cell line, two of them were females and one was non-DS male child with xeroderma pigmentosum. Three male and 11 female siblings were either normal or diagnosed as non-DS (SR = 0.27).

In the majority of the cases, paternal GM was inferred from the presence of trisomic cells in cultured blood cells. In two cases, mosaicism was detected in other tissue(s). A paternal origin of the extra chromosome 21 in the trisomic offspring was confirmed in four families, indicating a probable trisomy MI rescue in two carriers of GM and probable MII NDJ or postzygotic mitotic origin in one carrier; and in one case molecular analysis failed to define the mechanism resulting in T21 cell line.

Information on the maternal age at the time of delivery of their first affected offspring was available for 16 families. However three cases from a survey on prevalence of mosaicism in young parents (Additional File 2: Table S2, cases 3 - 5) were excluded from the analysis of maternal age. In this group, two in 13 mothers were aged 35 years and older.

### Total sample (all families with either maternal or paternal trisomy 21 gonadal mosaicism)

Overall, there were 61 families with maternal GM for T21 and 19 families with paternal GM for T21, indicating a strong female prevalence among carriers of GM. Among 80 families, there were reported 12 families showing mosaicism for T21 in successive generations. They included seven families with eight affected/miscarried carriers (Additional File 1: Table S1, cases 5, 26, 43, 46, 51, and Additional File 2: Table S2, cases 16 and 19), a family with one mosaic non-DS child (Additional File 2: Table S2, case 1), and four families with asymptomatic carriers of GM (Additional File 1: Table S1, cases 2, 3, 7, 61).

Among apparently transmitted cases of T21, there were more males than females among postnatally diagnosed individuals (66 M/44 F, SR = 1.5), no significant male prevalence among prenatally diagnosed cases (13 M/12 F, SR = 1.08) and female excess among miscarried fetuses (2 M/4 F, SR = 0.5). However, because of the limited sample, the difference does not reach statistical significance.

Nine of 134 (6.7%) individuals with confirmed T21 were mosaics for a normal cell line. In this group, there were one male with no clinical features of DS (Additional File 2: Table S2, case 1) and eight affected females (Additional File 1: Table S1, cases 5, 26, 43, 46, 51, and Additional File 2: Table S2, cases 16 and 19). The male to female ratio in this group is different from both the SR = 1.06 in the general population and from SR = 1.3 typical for T21 patients (p = 0.0158 and p = 0.0071, respectively). Therefore, the proportion of mosaics among affected females (8/59 = 14%) differs significantly from 0, the proportion found among 76 affected males (p = 0.005).

Of 14 families where the origin of the T21 in the GM carrier was traced, trisomy MI rescue was suggested as a mechanism of formation of parental GM in 78% (11/14). Rescue of trisomy MII or postzygotic mitotic origin were suggested in three cases. In four families with mosaicism in successive generations, the T21 in the carrier was most probably the result of the rescue of transmitted trisomy.

Mothers were young in either parental group, and mean maternal age in the combined group was 26.4 yr, with proportion of mothers aged 35 years and older being 5/65 = 8%. There were 51 families for which a sufficient reproductive history and pregnancy outcome were known. In 17 of these families, the first born DS child/fetus was not the first born child/fetus (Additional File 1: Table S1, cases 8, 10, 18, 20, 25, 27, 36, 38, 49, 51, 53, 59, 61, and Additional File 2: Table S2, cases 8, 9, 11, 14). In this group, the proportion of mothers aged 35 years and older at the time of DS birth was 6%, and mean maternal age was 28.4 yr. The analysis of grandmaternal age, however, showed a large proportion (9/23 = 39%) of them aged 35 yr and older at the time of the birth of their GM offspring.

## Discussion

### Mechanisms of germ mosaicism formation

Two different mechanisms are responsible for the formation of mosaicism. One is a mitotic error in a normal, euploid zygote resulting in a mosaic embryo having 46/47,+21 karyotype, the 45,-21 cell line being nonviable. Recently, it was demonstrated that mitotic errors of chromosome 21 are associated with non-viability of preimplantation embryo [24]. This most likely can be explained by the presence of nonvialble monosomic line. According to data on assessment mosaicism in human IVF embryo, > 3/8 of abnormal/removed blastomeres is considered detrimental for embryo survival [25].

The other mechanism is a NDJ in parental gametogenesis followed by an early postzygotic malsegregation of chromosome 21 ("trisomy rescue"). There are two mechanisms of chromosome malsegregation in a trisomic conceptus, chromosome loss and NDJ, the latter resulting in a mosaic embryo with a 46/47,+21/48,+21,+21 karyotype. *In vitro *studies in binucleated lymphocytes of trisomy 21 patients and of healthy children showed that the frequency of NDJ was significantly higher than the loss of chromosome 21. Moreover, malsegregation of chromosome 21 occurs more often in trisomic 21 cells than in disomic cells from normal children [26]. Although very rarely, some few cases of mosaicism for a tetrasomic line were found in prenatal samples [27].

It was suggested that the presence of a tetrasomic line in the gonads of a carrier of GM mosaicism might explain some cases of extraordinary occurrence of several conceptions of trisomy 21 in a one sibship [28-31]. It should be noted though, that no tetrasomic cells were reported in published studies on ovaries of carriers of GM [9,28,32-36]. Another underlying mechanism of a high recurrence of trisomic offspring in some families was proposed recently [4]. This mechanism suggests a specific type of secondary non-disjunction in a T21 oocyte by formation of either a trivalent or a bivalent plus univalent.

As stated in the recent review on somatic genome variation that addresses mosaic aneuploidy generation mechanisms [37], any disturbance occurring at molecular, supramolecular or intercellular level, can be associated with mitotic NDJ or other types of mitotic errors leading to aneuploidy. Among the most common causes of chromosome missegregation, different defects in kinetochore apparatus are suggested. Of them, merotelic kinetochore-microtubule attachment is considered as one of the commonest mechanism [37].

Up to now, there was lack of sufficient information on parental and cell division origin of trisomic line in asymptomatic GM carriers. Based on an estimation from maternal ages of the normal, mosaic and nonmosaic trisomic individuals, Richards [22] proposed that unlike mosaic DS, a large proportion of asymptomatic mosaic carriers started as normal zygotes. Since then, no studied were conducted for testing this hypothesis. In the present study, the majority of the mosaic parents were found to be a product of trisomic zygotes.

### Mosaicism transmission or genetic predisposition to nondisjunction

Surprisingly, the data collected give evidence for a high frequency of mosaic 46/47,+21 offspring in carriers of GM. In 80 studied families, seven families with eight affected mosaic females were identified. There is no appropriate explanation for the high prevalence of mosaic females among affected offspring of GM carriers. Both genetic predisposition to NDJ [11] and sex-specific rescue due to either sex-specific chromosome loss [38] or sex-specific selection against abnormal cell line [39] can be suggested.

Mosaicism for a normal line is not frequently found either in postnatally or in prenatally detected trisomics, accounting for only 1-3% of all free T21 cases [17,18,40]. In contrast to a significant male excess typical to DS individuals with non-mosaic T21 (SR~1.3), a notable female prevalence was reported for carriers of mosaicism, being higher in prenatally detected cases (SR = 0.6) than in newborns (SR = 0.95) [17,18,40]. Therefore, both the high frequency of the "inherited" mosaicism and the strong female prevalence among mosaic offspring of carriers of germ-line mosaicism found in the present study were not expected.

Five cases of unaffected carriers of mosaicism for T21 in two successive generations were also reported, including two families with asymptomatic mosaicism "inherited" by a healthy GM carrier [10,41], one family with non-DS mosaic child with xeroderma pigmentosum [11], and two nuclear families from a pedigree with recurrent T21 where several healthy family members were found to have uniparental disomy (UPD) of chromosome 21 [42]. The latter most probably indicates presence of GM in three generations, in the mother of two fetuses with T21 (case II/2), in her father (case III/1) and in his sister (case I/2), and in one of her paternal grandparents. Additionally, in some families where the individuals with T21 were second-degree and third-degree relatives, undetected GM could not be excluded [43]. One may suggest that the trisomic fetus in the family RDS-15 from the same study, who was identified as a product of meiosis II/mitotic NDJ of a paternal chromosome (*bbd*), inherited two chromosomes from his mosaic (*bc/bbc*) father, who in turn had inherited a trisomy from his mosaic mother (*ab/abb*), who also had given birth to twins with trisomy (*abc*). In the family RDS-19, the parents in whom NDJ in maternal meiosis I and in paternal meiosis II was identified were siblings. In this case, one may assume a grandmaternal GM (*ab/abb*) which was "transmitted" to her healthy mosaic daughter (*bc/bbc*) and to her son (*bc/bbc*) who gave birth to their offspring with non mosaic trisomy (*bcd *and *bbe*, respectively).

Summarizing, among 80 families, at least 12 families displaying mosaicism for T21 in successive generations were reported. There is most probably a bias towards publication of such cases. Higher numbers of families with trisomy 21 recurrence are needed for final evaluation of this phenomenon. Irrespective of the mechanisms of the mosaicism "transmission", this observation if confirmed in future studies, would indicate the relevance of cytogenetic testing of the unaffected offspring of GM carrier for the presence of mosaicism.

### Sex ratio in carriers of gonadal mosaicism

Whatever mechanism accounts for the loss of an extra chromosome 21 from a trisomic conceptus, the remarkable feature of GM carriers is the female prevalence. There is a four-fold female preponderance in two unbiased epidemiological studies in which parental transmission of the extra chromosome was reported (16 maternally transmitted and four paternally transmitted trisomy 21 cases) [33,44]. The proportion of female carriers of GM in this study (61 females and 19 males) is somewhat lower, probably because of a bias towards publishing "more interesting" cases of rare paternal transmission. Since corresponding impairment of spermatogenesis was not documented for carriers of GM for T21, some mechanisms other than reduced fertility in male carriers might be involved. Recent studies suggest that females may have GM for aneuploidy more often than males due to sex-specific chromosome loss in early embryogenesis [38].

### Sex ratio in affected offspring

A male excess in Down syndrome patients with non-mosaic T21 is a well known but poorly understood phenomenon. The sex ratio in this group varies in different populations, the average being close to 1.3. A meta-analysis of data from epidemiological studies suggests that the phenomenon is not restricted to free trisomy 21 alone but also appears in translocation T21, in carriers of mutant or inherited unbalanced translocations [15]. Several mechanisms were proposed to explain the skewed sex ratio in DS: meiotic disturbance (non-homologous co-orientation in male meiosis), fertilization event (greater accessibility of Y-bearing sperm to ova disomic for chromosome 21), and post-fertilization events (intrauterine selection against females).

Non-homologous co-orientation of the chromosome 21 and the X chromosome in male meiosis which was proposed as a meiotic mechanism of joint segregation of the chromosome 21 and the Y-chromosome [12,13,15] still needs more supporting data. Besides, this mechanism can only explain the male prevalence in T21 of paternal origin which constitutes a minor part of all T21 cases [45]. Data from the present study indicate that meiotic events may not be responsible for the skewed sex ratio in DS since similar male preponderance was found in both maternally- and paternally transmitted T21.

Fertilization mechanisms suggest that Y-bearing sperm can promote non-disjunction in the ova [15,16] or that a disomic ovum can be more readily fertilized by Y-carrying sperm [14]. However, both these mechanisms are not readily recognizable.

A comparison of the sex ratios in fetuses prenatally diagnosed with DS and in live births with DS suggests that there is no intrauterine selection favoring the survival of males [17,18]. However, prenatally detected cases represent a highly mixed group, including those referred for advanced maternal ages, fetal serum markers, ultrasound abnormalities, parental anxiety, etc. As to GM carriers, the predominant indication for prenatal testing was "having a previous child with Down syndrome".

The data presented in this paper suggest that a probable explanation of the male prevalence in DS is an intrauterine selection against female fetuses. Recent mFISH study demonstrated a strong female prevalence among chromosomally abnormal miscarried fetuses [46]. According to Kuo [47], the combined factors of embryonic quality and endocrine dysfunction in female carriers of GM might contribute to miscarriages that are very common to these patients. Unfortunately the sample of both prenatally detected and miscarried T21 offspring collected in the present study is too small to allow a definitive conclusion on the ability of mosaic mothers to carry affected fetuses to term depending on the gender of the fetus. It should be noted though that there is no significant male prevalence in the unaffected offspring of female carriers of GM (18 males and 15 females, SR = 1.2).

### Sex ratio in unaffected offspring

There is, however, a strong female excess among unaffected offspring of male carriers of GM, with 3 males and 11 females, the difference with unaffected offspring of female carriers of GM is significant, p = 0.037. Although the sample is small one might speculate about the genetic mechanism of such an unusual sex ratio. It might be a non homologous co-orientation of two chromosomes 21 with the X chromosome during the first meiotic division in the trisomic spermatocyte that leads to a preferential segregation of the X chromosome with one chromosome 21 and, consequently, to an excess of euploid females. However, a reciprocal strong excess of males among offspring with paternally derived T21, due to corresponding preferential segregation of the Y chromosome with two chromosomes 21, has not been observed. More families with paternal GM should be analyzed to confirm (or reject) this suggestion. Studies in sperm of male carriers might also contribute to understanding meiotic behavior of both the extra chromosome 21 and the sex chromosomes.

### Maternal and grandmaternal ages

Maternal age is a well-established risk factor for non mosaic regular T21. However although women of 35 years and older have an elevated risk of having trisomic offspring, a comparatively low proportion of all DS births occurs to these women. Approximately 60% of all DS cases are born to young parents [48]. The underlying mechanism is still unclear, but parental germ-line mosaicism is one likely factor.

Analysis of publications reporting maternal age composition worldwide from 1970 to 1995 showed that in most of them the mean maternal age in DS mothers was reported to be higher than 30, fluctuating from 29.5 in Taiwan to 35.6 in Libya. In the majority of that reports, mean population maternal age did not exceed 27, varying from 24.4 in Northeast Brasil to 28.1 year in Libya [16]. In the present study, the majority of mothers were young at the time of birth/conception of their first offspring with Down syndrome; the average maternal age was 26.4 years old which is similar to the average maternal age in the general population. One may argue that the mother's age, being the "age at first born DS child or fetus" could be biased lower as compared to average age of mothers in the population. However the figure of 26.4 is considerably lower compared to average age in DS mothers ascertained because of having first DS child. In the collected sample, there are 51 cases for which a sufficient reproductive history and pregnancy outcome were known. Of them, in 17 cases the first born DS child was not the first born child. In this group, the proportion of mothers aged 35 years or older at the time of the DS birth was 6%, and the mean maternal age was 28.4, which is still lower compared to average age in DS mothers.

In agreement with previous reports [20,49], grandmaternal age at the time when carriers of GM were born was significantly increased. This is consistent with the assumption that the trisomic line occurred frequently as a result of age-dependent meiotic error. GM carriers born to young parents probably start as diploid zygote followed by a mitotic NDJ. Postzygotic NDJ as a mechanism of T21 formation was proven not to be associated with advanced maternal age [50]. Unfortunately, the lack of information on both grandmaternal age and the nature of the trisomy in GM mosaicism carriers in the majority of the reported families did not allow analysis of the association between maternal age and the origin of trisomy in asymptomatic carriers of gonadal mosaicism.

## Materials and methods

The data for this study were obtained from an extensive literature review. Only cases that presented information sufficient to infer a high probability of true parental GM, together with known sex of the affected offspring, were included in the study. Because of the rarity of published cases, every effort was made to identify as many of the published cases as possible, including cases published in abstract form. A few cases were retrieved from log-books of St. Petersburg Centre of Medical Genetics (SPCMG) and Leningrad Region Medical Genetics Unit (LRMG). Whenever possible, information on the parental ages at the DS birth/conception and at the transmitting GM carrier's birth, as well as information on the sex of the unaffected offspring, was retrieved from the reported cases. Collected data were analyzed using binomial test and Chi-square test with Yates correction.

## Conclusion

Both direct (results of cytogenetic and molecular study of the origin of trisomic line) and indirect (advanced grandmaternal age on the side of GM carrier) evidences allow to assume that significant proportion of the mosaic parents had been conceived as trisomics. Female-specific trisomy rescue and genetic predisposition to postzygotic non-disjunction has been suggested as mechanisms of formation of both somatic mosaicism and GM. Typical male preponderance in affected nonmosaic offspring with either maternally- or paternally transmitted trisomy 21, indicates than meiotic events are not responsible for the skewed sex ratio in DS. However a female excess among unaffected offspring of male carriers of GM might be the result of meiotic non homologous co-orientation of chromosomes 21 and X in spermatogenesis.

## Competing interests

The author declares that they have no competing interests.

## Supplementary Material

Additional file 1 **Table S1. Details of families with maternal trisomy 21 gonadal mosaicism**. Tabular data presenting indication for the testing of the carrier for the presence of abnormal line, proportion of trisomic cell line; method of germ mosaicism ascertainment, maternal age at birth/conception of DS child/fetus, grandparental ages at birth of the carrier, outcome of carrier's pregnancies, and sex of both affected and unaffected offspring of female carriers of gonadal mosaicism. Click here for file

Additional file 2 **Table S2. Detailes of families with paternal trisomy 21 gonadal mosaicism**. Tabular data presenting indication for the testing of the carrier for the presence of abnormal line, proportion of trisomic cell line; method of germ mosaicism ascertainment, maternal age at birth/conception of DS child/fetus, grandparental ages at birth of the carrier, outcome of carrier's pregnancies, and sex of both affected and unaffected offspring of male carriers of gonadal mosaicism. Click here for file
