# Supplementary material for: Germ-line transmission of trisomy 21: Data from 80 families suggest an implication of grandmaternal age and a high frequency of female-specific trisomy rescue
Source: Mol Cytogenet. 2010 Mar 18;3:7. doi: 10.1186/1755-8166-3-7 (PMC2857837; doi:10.1186/1755-8166-3-7)
Supplement: Additional file 1 — Table S1. Details of families with maternal trisomy 21 gonadal mosaicism. Tabular data presenting indication for the testing of the carrier for the presence of abnormal line, proportion of trisomic cell line; method of germ mosaicism ascertainment, maternal age at birth/conception of DS child/fetus, grandparental ages at birth of the carrier, outcome of carrier's pregnancies, and sex of both affected and unaffected offspring of female carriers of gonadal mosaicism. [file 1755-8166-3-7-S1.DOC]

Table 1. Detailes of families with maternal trisomy 21 gonadal mosaicism

| Case  No | Source | Indication for the testing of the carrier for the presence of abnormal line | Proportion of trisomic cell line; method of germ mosaicism ascertainment | Maternal age at birth/conception of DS child/fetus, yr | | Grandparental ages at birth of the carrier (mat/pat), yr | Outcome of carrier's pregnancies | Trisomy 21 offspring with reported sex | | | | | | Comments |
| --- | --- | --- | --- | --- | --- | --- | --- | --- | --- | --- | --- | --- | --- | --- |
|  |  |  |  |  |  |  |  | Postnatal diagnosis | | Prenatal diagnosis | | Miscarriage | |  |
|  |  |  |  |  |  |  |  | male | female | male | female | male | female |  |
| 1 | Aarskog, 1969 [51] | recurrent T21 offspring | 5% BL | 22, 24 | | 39/45 | I and II - T21 child | 2 |  |  |  |  |  |  |
| 2 | Ban et al., 2003 [42] (family member II/3) | multiple T21 conceptions in the two nuclear families from the same pedigree | 0% BL, DNA studies, GM inferred from isoUPD21pat  (rescue of inherited trisomy) | 30, 32 | | ns | I and II - T21 fetus |  |  | 1 |  |  |  | Maternal father (family member 1/3) showed isoUPD21, suggesting mosaicism in three successive generations (grandgrandparental, grandpaternal, and maternal) |
| 3 | Ban et al., 2003 [42] (family member I/2, the aunt of the case II/3) |  | 0% BL, DNA studies, GM inferred from isoUPD21 (rescue of inherited trisomy) | ns | | ns | I - T21 child | 1 |  |  |  |  |  | The mother showed the isoUPD as her brother I/3, suggesting mosaicism in two generations |
| 4 | Blank et al., 1962 [52] | features of DS, child with T21 | 14% BL | 28 | | 40/42 | T21 child |  | 1 |  |  |  |  |  |
| 5 | Bruyere et al., 2000 [53] | study on parental origin of T21 in the mosaic child | 0% BL, DNA studies, GM (MII NDJ) inferred from UPD21 in the euploid line of the mosaic child | 22, 31 | | ns | I - T21 child, II - SA, III - T21 fetus, IV - 46,XX child |  | 1 | 1 |  |  |  | Mosaic 46/47,+21 female child, loss of the paternal chromosome in the euploid line |
| 6 | Butomo, Kovaleva, 1993 [29] (case 11) | recurrent T21 | 1.6% BL, GM (rescue of MI trisomy) proved by QFQ-polymorphism study | 29, 38, ns, ns | | ns | I - DS child, II - SA, III - T21 child, IV and V - T21 fetus | 1 | 1 | 2 |  |  |  | Clinical diagnosis in male child |
| 7 | Buzhievskaya et al., 1987 [41] (case 1) | ns | 5.8% 47,+21; 90.6% 47,+r(21)(p11;q11) BL | 26 | | ns | T21 child followed by normal female |  | 1 |  |  |  |  | Mosaicism in two successive generations (the mother and the maternal grandmother) |
| 8 | Cozzi et al., 1999 [36] | recurrent T21 conceptions | 0% BL, 70% ovulated oocytes, GM (mitotic NDJ) proved by DNA and cytogenetic markers | 32, ns, 36 | | ns | normal child followed by T21 child and two T21 fetuses | 1 |  |  |  |  |  |  |
| 9 | Cui et al., 2007 [54] | recurrent T21 | 0% BL, 4% SF, maternal origin of T21 in both conceptions confirmed by DNA analysis | 21, 27 | | ns | T21 child and T21 fetus |  | 1 |  | 1 |  |  |  |
| 10 | Dhadial and Pfeiffer, 1972 [55] | recurrent T21 | 5% BL, 0% SF | 21, 24 | | 27/40 | I - normal male, II - SA, III and IV - T21 child |  | 2 |  |  |  |  |  |
| 11 | English et al., 2001 [56]; personal communication, 2008 | recurrent T21 conceptions | 0% BL, DNA study result is consistent with the presence of GM (rescue of trisomy MI) | 20, 23 | | 21/24 | I - T21 fetus, II - normal female, III - T21 fetus, IV - 46,XY fetus |  |  | 1 | 1 |  |  |  |
| 12 | Ferrier, 1964 [57] (case 1) | ns | 28.6% BL, 21.6% SF | 25 | | 27/34 | I - T21 child | 1 |  |  |  |  |  |  |
| 13 | Fitzgerald et al., 1986 [58] | recurrent T21 offspring | 4% BL, 6% SF | 24, 26, 28 | | ns | I - T21 child, II and IV - T21 fetus, III - normal female | 1 |  |  |  |  |  |  |
| 14 | Frias et al., 2002 [59] (case P4) | study on prevalence of GM in young parents of DS children | 1.7% BL | 33 | | ns | three SA, T21 child |  | 1 |  |  |  |  |  |
| 15 | Fujita et al., 1992 [60] | multiple recurrence of T21 | 0% BL, 4-10% SF | ns, ns, 27 | | ns | two T21 children and T21 fetus | 1 |  |  | 1 |  |  |  |
| 16 | Gomirato-Sandrucci et al., 1971 [61] | recurrent T21 offspring | 5% ns | 19, 23 | | ns | two T21 children | 2 |  |  |  |  |  |  |
| 17 | Goodwin, Kousseff, 1986 [62] | recurrent T21 | 1%BL, 18% SF | 24, ns | | ns | two T21 children | 1 | 1 |  |  |  |  |  |
| 18 | Harris et al., 1982 [23] | recurrent T21 | 10% BL, 5% SF; maternal origin (mitotic NDJ or rescue of MII trisomy) proved by study of QFQ-polymorphism | under 30 | ns | | I - normal male, II-IV - T21 child | 2 | 1 |  |  |  |  |  |
| 19 | Ives et al., 1997 [30] | multiple recurrence of T21 | 8% BL, 5% SF, 100% cultured ovarian tissue, 0% oocytes | 21, ns….., 27 | 18 | | I-III - T21 child, IV-VII - T21 fetus, VIII-X - SA |  | 3 | 1 | 2 | 1 |  |  |
| 20 | Izakovic, Getlik, 1969 [63] | child with T21 | 21% BL | 27 | ns | | normal male and normal female followed by T21 child |  | 1 |  |  |  |  |  |
| 21 | James et al., 1998 [64] (family E) | multiple T21 pregnancies | 0.5% BL, 3% SF, DNA study revealed rescue of MI trisomy | 28, 32, 33 | ns | | I - DS child, II - normal male, III and IV - T21 fetus, V - 46,XX fetus |  | 1 | 1 | 1 |  |  |  |
| 22 | James et al., 1998 [64] (family F) |  | 4% BL | 23, 24, 29 | ns | | I - T21 child, II and IV - T21 fetus, III - normal male | 1 |  | 2 |  |  |  |  |
| 23 | Kaffe et al., 1974 [65] | recurrence of T21 | 22% BL | 25, 39 | >37 | | I - T21 child, II-IV normal child, V and VI - SA, VII - normal male, VIII - T21 child | 1 | 1 |  |  |  |  |  |
| 24 | Kovaleva, Tahmasebi-Hesari, 2007 [66] (family ZH) | study on parental origin of T21 in 151 consecutive families | 4% BL | 24, 28 | ns | | two DS children | 2 |  |  |  |  |  | Clinical diagnosis in one child |
| 25 | Kovaleva, Tahmasebi-Hesari, 2007 [66] (famuly UL) |  | 0.8% blood, GM (rescue of MI trisomy) confirmed by QFQ-polymorphism study | 24, 25, 28, 29 | 29/30 | | I and II - SA, III - normal male, IV – IA, V and VI - T21 child, VII and VIII - T21 fetus | 2 |  |  | 2 |  |  |  |
| 26 | Krishna Murthy, Farag, 1995 [67] (family 1) | multiple recurrence of T21 | 0.9% BL | 18, 19, 25 | ns | | I, II, and VI - T21 child, III - normal female, IV and V - SA | 2 | 1 |  |  |  |  | Mosaic 46/47,+21 female child |
| 27 | Krmpotik, Hardin, 1971 [68] | child with T21, multiple miscarriages | 10% BL | 33 | ns | | I - IX - three SA and six normal children, X - T21 child |  | 1 |  |  |  |  |  |
| 28 | Kuo, 2002 [47] (case 1) | study of 1,010 couples with a history of recurrent SA | 4% BL, 13% SF | 30 | ns | | I - 46,XX SA, II - T21 SA, III - 46,XY child |  |  |  |  | 1 |  |  |
| 29 | Kuo, 2002 [47] (case 2) |  | 8% BL, 5% SF, 100% cultured ovarian tissue, 0% oocytes | 26 | ns | | I and II - SA, III - T21 SA, IV - 46,XX child, V - 46,XY SA, VI - 45,XY, -21 SA |  |  |  |  |  | 1 |  |
| 30 | LRMG logbook (family DR) | child with T21, parental anxiety | low level, BL | 18 | ns | | T21 child, 46,XY child | 1 |  |  |  |  |  |  |
| 31 | LRMG logbook (family MI) | child with T21, parental anxiety | 1.4% BL | 28 | ns | | T21 child | 1 |  |  |  |  |  |  |
| 32 | LRMG logbook (family PR) | child with T21, parental anxiety | 8% BL | 24 | ns | | T21 child | 1 |  |  |  |  |  |  |
| 33 | Makino, 1964 [69] | search for GM in families with T21 | BL | 29 | ns | | T21 child | 1 |  |  |  |  |  |  |
| 34 | Mikkelsen et al., 1970 [70] | survey of patients with DS born to young mothers | 3% BL, 13% SF | 20, 29 | 22/28 | | I – T21 child, II – SA, III – T21 child | 1 | 1 |  |  |  |  |  |
| 35 | MRCC, 1977 [71] (case 342) | ns | 9% BL, 22% and 84% ovaries | ns | ns | | T21 fetus |  |  |  | 1 |  |  |  |
| 36 | Nielsen et al., 1988 [28] | multiple recurrence of T21 | 0% BL and SF, 15% ovary, rescue of MI trisomy revealed by QFQ polymorphism study | 23, 25, 26, 27, 28, 30 | ns | | I - male with no DS features, died, II and V - DS child, IV and VI - T21 child, III - SA, VII and IX - T21 fetus, VIII, X, and XI - IA | 2 | 2 | 1 | 1 |  |  | Clinical diagnosis in one male child and in stillborn girl |
| 37 | Nuzzo et al., 1975 [72] | recurrent T21 | 0% BL, 0.9% SF | 30, 32 | 29 | | I - T21 child, II - T21 twins | 2 |  |  |  |  |  | Twins as single entry |
| 38 | Osuna, Moreno, 1977 [73] | multiple recurrence of T21 | 6% BL | 24, 27, 29 | 26/23 | | normal male followed by three T21 children | 1 | 2 |  |  |  |  |  |
| 39 | Pangalos et al., 1992 [43] (family RDS-09) | study on origin of T21 in families with recurrence of T21 | 2% BL, maternal origin (rescue of MI trisomy) confirmed by DNA analysis | 26, 29 | ns | | I and II - T21 child |  | 2 |  |  |  |  |  |
| 40 | Pangalos et al., 1992 [43] (family RDS-10) |  | 2% BL, maternal origin confirmed by DNA analysis | 28, 32 | ns | | I - T21 child, II -SA, III - normal female, IV - T21 fetus | 1 |  | 1 |  |  |  |  |
| 41 | Pangalos et al., 1992 [43] (family RDS-13) |  | 0% BL, GM (rescue of MI trisomy) inferred from DNA analysis | 25, 29 | ns | | I and III - T21 child, II - normal female | 1 | 1 |  |  |  |  |  |
| 42 | Pangalos et al., 1992 [43] (family RDS-14) |  | 0% BL, GM (rescue of MI trisomy) inferred from DNA analysis | 36, 38 | ns | | I and II - T21 fetus |  |  | 1 | 1 |  |  |  |
| 43 | Parke et al., 1980 [9] | poor reproductive history, child with T21 | 2% BL, 0% SF, 6% ovarian fibroblastic cells | 24 | ns | | I-III - SA, IV - T21, V and VI - normal 46,XX child |  | 1 |  |  |  |  | Mosaic 46/47,+21 female child |
| 44 | Raichs and Tamparillas, 1967 [74] | child with T21 | 20% BL | 27 | 36 | | T21 child | 1 |  |  |  |  |  | Mosaic 46,XY,i(121q)/47,XY,+21 |
| 45 | Rowe et al., 1989 [75] | Alzheimer disease, child with T21 | 10% ns | 26 | ns | | T21 child |  | 1 |  |  |  |  |  |
| 46 | Sachs et al., 1990 [34] (family B) | multiple recurrence of T21 | 3% BL, 14% SF, 44% and 47% ovaries | ns | ns | | I - T21 child, II, VI, and VII - T21 fetus, III-V - SA, VIII - 46,XY fetus |  | 1 |  | 1 |  | 2 | Two mosaic 46/47,+21 miscarried female fetuses |
| 47 | Smith et al., 1962 [76] | recurrent T21 | 27% BL, 75% SF | ns, 19 | ns | | two T21 children | 2 |  |  |  |  |  |  |
| 48 | SPCMG logbook (family BR) | poor reproductive history, child with T21 | 15% BL | 29 | ns | | I, III, and IV - IA, II - mole, V - T21 child, VI - 46,XY fetus |  | 1 |  |  |  |  |  |
| 49 | SPCMG logbook (family PA) | child clinically diagnosed as DS, dead | 2% BL | 28 | ns | | I - normal female, II-IV - IA, V - child with DS | 1 |  |  |  |  |  | Clinical diagnosis |
| 50 | SPCMG logbook  (family SH) | child with T21, parental anxiety | 4% BL | 26 | ns | | T21 child | 1 |  |  |  |  |  |  |
| 51 | SPCMG logbook (family SO) | child with T21, parental anxiety | 4% BL | 38 | ns | | I-IV - IA, V - normal female, VI - T21 child |  | 1 |  |  |  |  | Mosaic 46/47,+21 female child |
| 52 | SPCMG logbook (family UV) | poor reproductive history, child with T21 | BL | 41 | ns | | three SA, T21 child |  | 1 |  |  |  |  |  |
| 53 | Street et al., 2007 [77], personal communication, 2009 | previous pregnancy with T21 | 1.5% BL; T21 maternal cell contamination of an amniotic fluid specimen from a normal male pregnancy | 33 | ns | | I and II - SB and normal livebirth, III - SA, IV - T21 fetus, V - normal 46,XY child |  |  | 1 |  |  |  |  |
| 54 | Sutherland et al., 1972 [78] | multiple resurrence of T21, some stigmata of DS | 0% BL, 0% bone marrow; 6% further BL culture | ns | ns | | three T21 children | 3 |  |  |  |  |  |  |
| 55 | Taylor, 1970 [32] | child with T21 | 6% BL, 6% SF; 89% and 92% in fibroblasts-like cells from ovaries | ns | ns | | T21 child | 1 |  |  |  |  |  |  |
| 56 | Timson et al., 1971 [79] | ns | 10% BL | 26 | ns | | T21 child followed by normal 46,XX | 1 |  |  |  |  |  |  |
| 57 | Tseng et al., 1994 [35] | multiple recurrence of T21 | 0% BL, 40% right ovary | 22, 23, 27 | ns | | I - T21 child, II - DS child, III - T21 child, IV and V - IA, VI - normal 46,XY child | 1 | 2 |  |  |  |  | Clinical diagnosis in the female child |
| 58 | Uchida, Freeman, 1985 [33] (family 6) | study on parental mosaicism in a random series of 374 families | 2% BL | 27, 29, 31 | ns | | three T21 children | 3 |  |  |  |  |  |  |
| 59 | Verresen et al., 1964 [80] | ns | 10% BL | 30 | 34 | | normal male, T21 child | 1 |  |  |  |  |  |  |
| 60 | Weinstein, Warkany, 1963 [81] | child with T21 | 17% BL, 18% SF | 17 | 39 | | I - T21 child | 1 |  |  |  |  |  |  |
| 61 | Werner et al., 1982 [10] | child with T21, some stigmata of DS | 31% BL, rescue of MI trisomy revealed by QFQ polymorphism | 31 | 43/46 | | I and IV - SA, II - normal male, III - T21 child, | 1 |  |  |  |  |  | Mosaicism in two successive generations (the mother and the maternal grandmother) |
| ***All maternally derived cases, n*** | | | | | | | ***19 males and 16***  ***females unaffected*** | ***50*** | ***34*** | ***13*** | ***12*** | ***2*** | ***3*** | ***6 /108=5.6 % affected individuals/fetuses with 46/47,+21 mosaicism*** |
| ***Sex ratio*** | | | | | | | ***1.2*** | ***1.5*** | | ***1.08*** | | ***0.67*** | |  |

BL: blood sample

IA: induced abortion

QFQ polymorphism: chromosome 21 short arm polymorphism identified by QFQ-banding technique

SA: spontaneous abortion

SB: stillborn

SF: skin fibroblasts
