# Supplementary material for: Germ-line transmission of trisomy 21: Data from 80 families suggest an implication of grandmaternal age and a high frequency of female-specific trisomy rescue
Source: Mol Cytogenet. 2010 Mar 18;3:7. doi: 10.1186/1755-8166-3-7 (PMC2857837; doi:10.1186/1755-8166-3-7)
Supplement: Additional file 2 — Table S2. Detailes of families with paternal trisomy 21 gonadal mosaicism. Tabular data presenting indication for the testing of the carrier for the presence of abnormal line, proportion of trisomic cell line; method of germ mosaicism ascertainment, maternal age at birth/conception of DS child/fetus, grandparental ages at birth of the carrier, outcome of carrier's pregnancies, and sex of both affected and unaffected offspring of male carriers of gonadal mosaicism. [file 1755-8166-3-7-S2.DOC]

Table 2. Detailes of families with paternal trisomy 21 gonadal mosaicism

| Case  No | Source | Indication for the testing of the carrier for the presence of abnormal line | Proportion of trisomic cell line; method of germ mosaicism ascertainment | Maternal age at birth/conception of DS child/fetus, yr | Grandparental ages at birth of the carrier (mat/pat), yr | Outcome of carrier's pregnancies | Trisomy 21 offspring with reported sex | | | | | | Comments |
| --- | --- | --- | --- | --- | --- | --- | --- | --- | --- | --- | --- | --- | --- |
|  |  |  |  |  |  |  | Postnatal diagnosis | | Prenatal diagnosis | | Miscarriage | |  |
|  |  |  |  |  |  |  | male | female | male | female | male | female |  |
| 1 | Casati et al., 1992 [11] | chromosome fragility study in patients with xeroderma pigmentosusm | 2% BL, 0% SF; paternal origin (rescue of MII trisomy or mitotic NDJ) confirmed by study of QFQ polymorphism | 28/29 | 41/47 | T21 child followed by normal male | 1 |  |  |  |  |  | Mosaic 46/47,+21 non-DS male child with xeroderma pigmentosum |
| 2 | Domany, Metneki, 1976 [82] (case L.K.) | child clinically diagnosed as DS, dead | 15% BL | 27/27 | ns | I - T21 child | 1 |  |  |  |  |  |  |
| 3 | Frias et al., 2002 [59] (case P19) | study on prevalence of GM in young parents of DS children | 1.5% BL | 24/33 | ns | T21 child |  | 1 |  |  |  |  |  |
| 4 | Frias et al., 2002 [59] (case P24) |  | 1.3% BL | 25/26 | ns | two T21 children, SB malformed male, normal female | 1 | 1 |  |  |  |  |  |
| 5 | Frias et al., 2002 [59] (case P25) |  | 1.5% BL | 25/25 | ns | two T21 children, two SA, normal female, unaffected SB female | 1 | 1 |  |  |  |  |  |
| 6 | Hsu et al., 1971 [83] (family D) | child with T21 | 6% BL | 22/21 | 30/40 | I - T21 child | 1 |  |  |  |  |  |  |
| 7 | Hsu et al., 1971 [83] (family K) | child with T21 | 4.6% BL, 4% SF | 28/30 | 19/22 | I - T21 child | 1 |  |  |  |  |  |  |
| 8 | Hsu et al., 1971 [83] (family N) | recurrent T21 | 0% BL, 7.5% SF, 4% testicular biopsy fibroblast culture, 60% spermatogonial metaphases | 19/23, 21/25 | 35/45 | I - normal female, II and III - T21 child | 1 | 1 |  |  |  |  |  |
| 9 | Kovaleva, Tahmasebi-Hesari, 2007 [66] (family A-K) | study on parental origin of T21 in 151 consecutive families | 0% BL, rescue of MI trisomy revealed by study of QFQ polymorphism | 32/29 | 27/28 | I - normal female, II - IA, III - T21 child | 1 |  |  |  |  |  |  |
| 10 | Kovaleva, Tahmasebi-Hesari, 2007 [66] (family ST) |  | 4% blood | 24/23 | 32/34 | I - T21 child | 1 |  |  |  |  |  |  |
| 11 | Kovaleva and Tahmasebi-Hesari, 2007 [66] (family V) |  | 1% blood, rescue of MI trisomy revealed by study of QFQ polymorphism | 31/38 | ns | I - normal female, II - IA, III - T21 child | 1 |  |  |  |  |  |  |
| 12 | Massimo et al., 1967 [83] Mattei et al., 1974 [84] | ns | 21.5% blood | 35/ns | 30 | two T21 children | 1 | 1 |  |  |  |  |  |
| 13 | Mehes, 1973 [85] | child with T21 | 7% blood | 22/23 | 31/36 | I - T21 child | 1 |  |  |  |  |  |  |
| 14 | Pangalos et al., 1992 [43] (family RDS-02) | study on the origin of T21 in families with recurrence of T21 | 2% blood, paternal origin of T21 confirmed by DNA analysis | 28/34, 34/40 | ns | I - normal male, II, III, and V - normal female, IV and VI - T21 | 1 | 1 |  |  |  |  |  |
| 15 | Papp et al., 1974 [86] | child with T21 | 6% blood | 19/30 | 33/32 | I - T21 child, II - normal female |  | 1 |  |  |  |  |  |
| 16 | Sachs et al., 1990 [34] (family A) | multiple recurrence of T21 | 22% blood | ns | ns | I and IV - SA, II - T21 child, III - T 21 fetus, V - T21 SA, VI - 46,XX fetus |  | 1 |  | 1 |  | 1 | Mosaic 46/47,+21 miscarried female fetus |
| 17 | Soltan et al., 1964 [87] | study on families with T21 recurrence | % ns BL | 38/32, 41/35 | 41/35 | two DS children | 2 |  |  |  |  |  | Clinical diagnosis in one child |
| 18 | De Toni et al., 1967 [88] | recurrent T21 | ns | ns | ns | two T21 children, no healthy children | 1 | 1 |  |  |  |  |  |
| 19 | Walker, Ising, 1969 [89] | DS features, child with T21 | 30% blood | ns | ns | T21 child |  | 1 |  |  |  |  | Mosaic 46/47,+21 female child |
| ***All paternally derived cases, n*** | | | | | | ***3 ales and 11***  ***females unaffected*** | ***16*** | ***10*** | ***0*** | ***1*** | ***0*** | ***1*** | ***2/27=7.2 % affected child/fetus with 46/47,+21 mosaicism*** |
| ***Sex ratio*** | | | | | | ***0.27*** | ***1.6*** | |  | |  | |  |

BL: blood sample

IA: induced abortion

QFQ polymorphism: chromosome 21 short arm polymorphism identified by QFQ-banding technique

SA: spontaneous abortion

SB: stillborn

SF: skin fibroblasts
